# Transarterial Chemoembolization Combined With Radiofrequency Ablation *Versus* Repeat Hepatectomy for Recurrent Hepatocellular Carcinoma After Curative Resection: A 10-Year Single-Center Comparative Study

**DOI:** 10.3389/fonc.2021.713432

**Published:** 2021-09-09

**Authors:** Xin Zheng, Yanqiao Ren, Hanqing Hu, Kun Qian

**Affiliations:** ^1^Department of Hepatopancreatobilary Surgery, The First College of Clinical Medical Sciences, China Three Gorges University, Yichang, China; ^2^Department of Radiology, Union Hospital, Tongji Medical College, Huazhong University of Science and Technology, Wuhan, China; ^3^Hubei Province Key Laboratory of Molecular Imaging, Wuhan, China

**Keywords:** recurrent hepatocellular carcinoma, transarterial chemoembolization, radiofrequency ablation, repeat hepatectomy, overall survival, progression-free survival

## Abstract

**Background:**

The purpose of this study was to compare the efficacy and safety of transarterial chemoembolization (TACE) in combination with radiofrequency ablation (RFA) (TACE-RFA) and repeat hepatectomy in the treatment of recurrent hepatocellular carcinoma (HCC) after curative resection.

**Methods:**

This retrospective study evaluated consecutive medical records of patients who received either TACE-RFA or repeat hepatectomy between January 2010 and May 2021. Overall survival (OS), progression-free survival (PFS), and complications were compared.

**Results:**

Of the 2672 patients who received either TACE-RFA or repeat hepatectomy, 111 eligible patients were included in our study, 63 in the TACE-RFA group and 48 in the repeat hepatectomy group. The median OS was 38 months in the TACE-RFA group and 42 months in the repeat hepatectomy group, with no statistically difference between the two groups (*P*=0.45). Meanwhile, there was also no statistically significant difference in PFS between the two groups (*P*=0.634). Although both groups achieved similar outcomes, the rate of major complications was significantly higher in the repeat hepatectomy group (*P*=0.003).

**Conclusions:**

Patients with recurrent HCC in the TACE-RFA group and the repeat hepatectomy group had similar OS and PFS regardless of the patient’s tumor diameter, but the TACE-RFA group was safer and more minimally invasive.

## Introduction

Hepatocellular carcinoma is the most frequent liver cancer, and liver cancer is the fifth most common cancer and the second most common cause of cancer-related death worldwide (1). Curative hepatectomy is one of the best first-line treatments for specific patients. Median survival after curative hepatectomy for HCC patients has been reported to be 50-70 months (2–4). However, the presence of intrahepatic recurrence and *de novo* tumor in the residual liver after curative hepatectomy is common, with a reported 5-year recurrence rate as high as 70%-80% (5). In addition, cirrhosis, tumors larger than 5 cm in diameter, positive histological margins, or portal vein invasion has been demonstrated to be potential risk factors for recurrence (6, 7). Although this is a common clinical manifestation, there are still no clear global algorithms or guidelines on the management of recurrent HCC after hepatectomy, which remains a thorny issue that currently confounds clinicians and patients.

For recurrent HCC, repeat hepatectomy or salvage liver transplantation may be the best treatment. Repeat hepatectomy is reported to be an effective and safe treatment option (8–10). However, surgical treatment is not indicated for most of these patients because of limited reserve of liver function in the residual liver, intrahepatic multiple recurrences, postoperative adhesion, or lack of a liver donor (11, 12). Therefore, only a few patients benefit from curative treatments, which may create an incentive to explore other therapies and methods.

Transarterial chemoembolization (TACE), which combines targeted chemotherapy with arterial embolization, is a well-tolerated procedure with limited hepatotoxicity and is effective in patients with recurrent HCC with borderline liver function (13, 14). However, it has been reported that TACE alone is difficult to cause complete tumor necrosis even if the tumor diameter is small (15, 16). It has been reported that the combination of TACE and radiofrequency ablation (RFA) has the following theoretical advantages (17, 18): (1) TACE can reduce the heat sink effect, thereby increasing the ablation range; (2) Satellite lesions can be detected through TACE, which is more beneficial to RFA. As described by the theoretical advantages, many studies (19, 20) have also reported satisfactory effects of TACE combined with RFA (TACE-RFA) in the treatment of HCC.

Currently, there are studies (21, 22) comparing the efficacy of surgical resection and TACE in the treatment of recurrent HCC, and there are also studies (23, 24) comparing the efficacy of surgical resection and RFA in the treatment of recurrent HCC. However, to our knowledge, there are few reports on the efficacy of repeat hepatectomy and TACE-RFA in the treatment of recurrent HCC after resection. Thus, the purpose of this retrospective study was to compare the efficacy and safety of TACE-RFA and repeat hepatectomy in the treatment of recurrent HCC. In addition, Peng et al (25) concluded that TACE-RFA had a similar effect to hepatectomy for recurrent HCC with a diameter of < 5cm, but for recurrent HCC with a tumor diameter of > 5cm, it has not been reported so far. Hence, another purpose of this study was to investigate the efficacy of TACE-RFA and hepatectomy for recurrent HCC with a diameter of more than 5cm.

## Materials and Methods

### Study Design and Patient Selection

This retrospective comparative study was approved by the local hospital ethic committee. Written informed consent was obtained from all patients prior to treatment.

From January 2010 to May 2020, 2672 patients with recurrent HCC after hepatectomy were admitted to our hospital. Before these patients were treated, the treatment strategy was recommended by the multidisciplinary oncology committee. Repeat hepatectomy was recommended based on the same criteria as initial resection, including Child-Pugh class A patients with solitary or oligonodular (2-3 nodules < 3cm) recurrence, preserved liver function, and sufficient liver volume (the residual liver volume after repeat hepatectomy must be more than 40% of the standard liver volume) without severe portal hypertension. TACE-RFA was considered in patients with Child-Pugh class A or B, no vascular involvement, and no severe ascites, and when repeated hepatectomy was not possible due to insufficient hepatic reserve function. Meanwhile, the time of RFA after TACE depends on the disappearance of complications and recovery of liver function after embolization. In our center, RFA is usually performed 1-2 weeks after TACE.

The diagnosis of recurrent HCC was based on the diagnostic criteria of the European Association for the Study of Liver (EASL) and the American Association for the Study of Liver Disease (26). A total of 111 consecutive patients who received either TACE-RFA (n=63) or repeat hepatectomy (n=48) meeting the following inclusion criteria were enrolled in the study: (1) first intrahepatic recurrence after the curative resection; (2) Child-Pugh class A or B; (3) no evidence of invasion into the macroscopic vascular, extrahepatic metastasis, or uncontrolled ascites; (4) an Eastern Cooperative Oncology Group (ECOG) performance status of 0 and expected survival of >3 months; (5) patients who refuse to undergo liver transplantation. The patient was excluded if the exclusion criteria were met: (1) had previously received any treatment for recurrent HCC; (2) hepatic dysfunction (total bilirubin serum >3 mg/dL, serum albumin level <2.0 mg/dL, INR > 1.5), renal impairment (serum creatinine level >2mg/dL); (3) uncontrolled infection.

### TACE

Conventional transarterial chemoembolization (cTACE) was performed by two experienced interventional radiologists according to our institutional standard protocol (19, 27). Briefly, in all TACE procedures, angiography of the celiac trunk and superior mesenteric artery was performed to visualize the arterial vascularization of the liver and to evaluate portal vein patency. The epirubicin-lipiodol emulsion, which prepared by dissolving 60 mg/m2 of epirubicin in 1–2 ml of a 2% lidocaine, before mixing with 5–20 ml lipiodol was delivered directly into the feeding artery under fluoroscopic guidance, after placing the catheter tip in the distal feeding arteries as close to the tumor as possible using either the standard 5 Fr catheter or a 3 Fr coaxial catheter when necessary, followed by the injection of 300-500um gelatin sponge particles. The endpoint of embolization was the tumor vessels were completely filled with the drugs and the tumor stain disappeared on angiographic imaging.

### RFA

The RFA procedure was performed in accordance with the standard treatment regimen described in our previous study (19). In short, percutaneous RFA was performed using a RITA 1500 generator (RITA Medical Systems, Mountain View, CA, USA) under real-time ultrasound and or both CT guidance, and different needle electrodes were used as follows: For tumors <= 2.0 cm in diameter, a single extendable electrode was used; otherwise, a multi-electrode was used. And to accomplish a safe range of 0.5–1.0 cm, multiple overlapping ablation zones were demanded. Single or multiple overlapping ablations were performed to achieve an ablation zone with at least a 0.5–1.0 cm ablative margin around the tumor. After the RFA procedure, the intrahepatic needle track was cauterized during electrode retraction to prevent bleeding or tract seeding.

### Repeat Hepatectomy

The liver function was evaluated by Child-Pugh scoring system before repeat hepatectomy. Among all patients who underwent repeat hepatectomy, 42 patients (87.5%) were Child-Pugh A stage and 6 patients (12.5%) was Child-Pugh B stage. Due to the serious abdominal adhesion during the repeat operation and in order to minimize the occurrence of complications, we chose an open operation. Patients were informed of the risks of the surgery before consent for the operation was obtained. Surgical resection was carried out in a standard procedure by a surgical team consisting of three experienced surgeons who had more than 10 years of experience in hepatectomy. The operating procedure is briefly as the liver is accessed by a right subcostal incision with midline extension, followed by intraperitoneal exploration to exclude disseminated disease. After initial mobilization of the falciform ligament, the liver is fully separated from the triangular and coronary ligament connecting the liver and diaphragm. Intraoperative ultrasound within the parenchyma localizes all suspected tumor nodules and identifies the portal vein and the liver veins. After this, the first porta hepatis occlusion band was preset, the portal vein was dissected, and the portal vein branch of the hepatic segment where the tumor was located was blocked. Then pre-excision line was marked according to the ischemia line, and the liver was cut by ultrasonic scalpel and bipolar cautery, then test with lipofundin is performed by retrograde flushing over the remaining cystic duct and obstruction of the main hepatic duct to detect and close the bile leakage at the transection surface. The transection surface is hemostased by coagulation with an argon beamer and bipolar cautery.

### Definition and Evaluation of Data

Overall survival (OS) referred to the interval between the first TACE procedure or repeat hepatectomy and the date of death or last follow-up. Progression-free survival (PFS) was known as the period between the date of the first TACE procedure or repeat hepatectomy and the date of progression for patients who displayed radiologic evidence of disease progression or the date of death. Complications or side effects were evaluated according to the Common Terminology Criteria for Adverse Events (version 5.0). Major complications were events leading to death and disability, which increase the level of care, or result in hospital admission, or substantially prolong the length of hospitalization (28).

### Follow-Up

All patients were followed up until May 2021. Patients in both groups were evaluated 4 to 6 weeks after treatment. Reexamination included laboratory tests (hematology and biochemical markers) and abdominal contrast-enhanced CT or magnetic resonance (MR). CT or MR imaging at 4-6 weeks after initial treatment were compared with preoperative imaging, and objective tumor radiologic regression (ORR) and disease control rate (DCR) were determined in both groups according to the Modified Response Evaluation Criteria in Solid Tumors (mRECIST) (29). ORR referred to complete response (CR) or partial response (PR). DCR represented CR, PR or stable disease (SD). During the follow-up period, tumor recurrence was divided into local recurrence, intrahepatic recurrence and extrahepatic metastasis. Local recurrence is defined as the presence of tumors in or around the primary lesion. Intrahepatic recurrence refers to the new lesion being more than 2.0 cm away from the primary lesion. Extrahepatic metastasis refers to extrahepatic tumor lesions. When residual viable HCCs or recurrent tumors, intrahepatic distant metastasis or extrahepatic metastasis occurred during the follow-up period, patients were given corresponding treatments such as resection, RFA, TACE, sorafenib and conservative treatment according to the characteristics of tumor recurrence, liver function status and patient requirements. Imaging (contrast-enhanced CT or MR) and laboratory examinations were performed every 2-3 months and patients were followed up until death or the end of the study’s follow-up.

### Statistical Analyses

SPSS software (Version 24.0; IBM, Armonk, New York) was used for all statistical analyses, and *P* < 0.05 indicated a statistically significance. Discrete variables were represented by numbers with percentages were calculated by Chi-square test, and continuous variables were presented as mean ± standard deviation. Kaplan-Meier method was used to evaluate the differences in OS and PFS between the two groups. The 95% confidence interval (CI) was calculated for median OS, median PFS, and hazard ratio (HR). Log-rank test was used for univariate analysis, in which variables with *P* value less than 0.10 in univariate analysis were added to multivariate analysis. Potential prognostic variables affecting OS and PFS were calculated using a Cox proportional hazard regression model. 

## Results

### Study Population and Patient Characteristics

**Figure 1:**
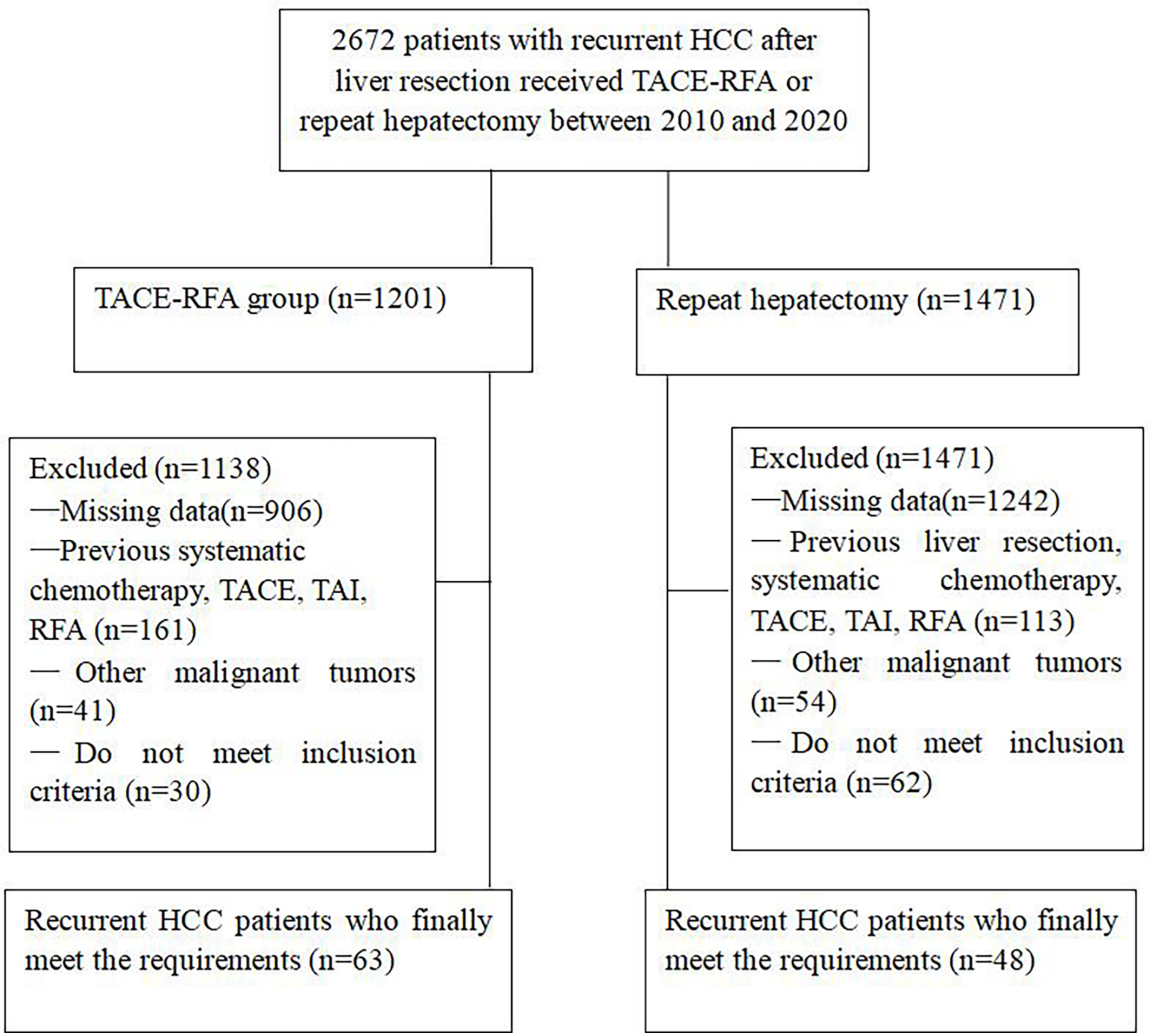
Flow chart shows the screening procedure for recurrent HCC patients after curative resection.

From January 2010 to May 2020, a total of 2672 patients received TACE-RFA or repeat hepatectomy, and 2561 patients were excluded because they did not meet the research requirements, as shown in **Figure 1**. Finally, a total of 111 recurrent HCC patients were enrolled in this study, 63 of whom received TACE-RFA and 48 of whom received repeat hepatectomy. There were 55 males (87.3%) and 8 females (12.7%) in the TACE-RFA group, with an average age of 53.1 ± 12.7 years old. There were 39 males (81.3%) and 9 females (18.7%) in the repeat hepatectomy group, with an average age of 52.0 ± 12.5 years old. There was no significant difference in baseline data between the two groups (**Table 1**).

**Table 1:** Baseline characteristics.

Characteristics	TACE-RFA (N=63) (No, %; Mean ± SD)	Repeat hepatectomy (N=48) (No, %; Mean ± SD)	*P* value
**Gender**			0.38
Male	55 (87.3%)	39 (81.3%)	
Female	8 (12.7%)	9 (18.7%)	
**Age (years)**	53.1 ± 12.7	52.0 ± 12.5	0.63
**Bilirubin (µmol/L)**	16.8 ± 8.3	17.9 ± 13.9	0.60
**Albumin (g/L)**	38.2 ± 5.4	38.6 ± 4.8	0.65
**PT(s)**	14.1 ± 1.4	14.3 ± 1.9	0.55
**AST (µmol/L)**	38.9 ± 17.4	34.6 ± 13.9	0.17
**ALT (µmol/L)**	35.7 ± 18.0	33.0 ± 15.8	0.42
**Tumor size**	4.0 ± 3.0	3.9 ± 2.2	0.86
**Tumor number**	1.48 ± 0.97	1.38 ± 0.64	0.53
**Hepatitis**			0.91
Hepatitis B	52 (82.5%)	40 (83.3%)	
Other	11 (17.5%)	8 (16.7%)	
**α-Fetoprotein level**			0.24
>400 ng/mL	28 (44.4%)	16 (33.3%)	
≤400 ng/ml	35 (55.6%)	32 (66.7%)	
**Child-Pugh score**			0.82
A	56 (88.9%)	42 (87.5%)	
B	7 (11.1%)	6 (12.5%)	
**BCLC**			0.14
A	39 (61.9%)	36 (75.0%)	
B	24 (38.1%)	12 (25.0%)	
**Interval of recurrence** **from initial treatment** **(months)**	22.5 ± 19.4	22.1 ± 19.5	0.93

TACE, Transcatheter arterial chemoembolization; RFA, Radiofrequency ablation; SD, Standard deviation; PT, Prothrombin time; AST, Aspartate aminotransferase; ALT, Alanine aminotransferase; BCLC, Barcelona Clinic Liver Cancer.

The median follow-up period was 34 months (range, 4–106 months) in the TACE-RFA group and 30.5 months (range, 0–92 months) in the repeat hepatectomy group. In the TACE-RFA group, 48 (76.2%) patients died during the observation period, and in the repeat hepatectomy group, 29 (60.4%) patients died.

### Treatment Response and Recurrence

The morphologic response of target lesions was verified using abdominal contrast-enhanced CT or MR imaging. In the TACE-RFA group, 17 patients achieved CR, 25 patients achieved PR, and 11 patients achieved SD. Hence, the ORR and DCR in the TACE-RFA group were 66.7% and 84.1%, respectively. Meanwhile, during the period of follow-up, in the TACE-RFA group, a total of 43 patients (68.3%) had recurrence, including 11 patients (17.5%) with local recurrence, 25 patients (39.7%) with intrahepatic recurrence, 7 patients (11.1%) with extrahepatic metastases, and a total of 30 patients (62.5%) had recurrence in the repeat hepatectomy group, including 6 patients (12.5%) with local recurrence, 19 patients (39.6%) with intrahepatic recurrence, 5 patients (10.4%) with extrahepatic metastases. There was no significant difference in recurrence rate between the two groups (*P*=0.527).

### Overall Survival

**Figure 2:**
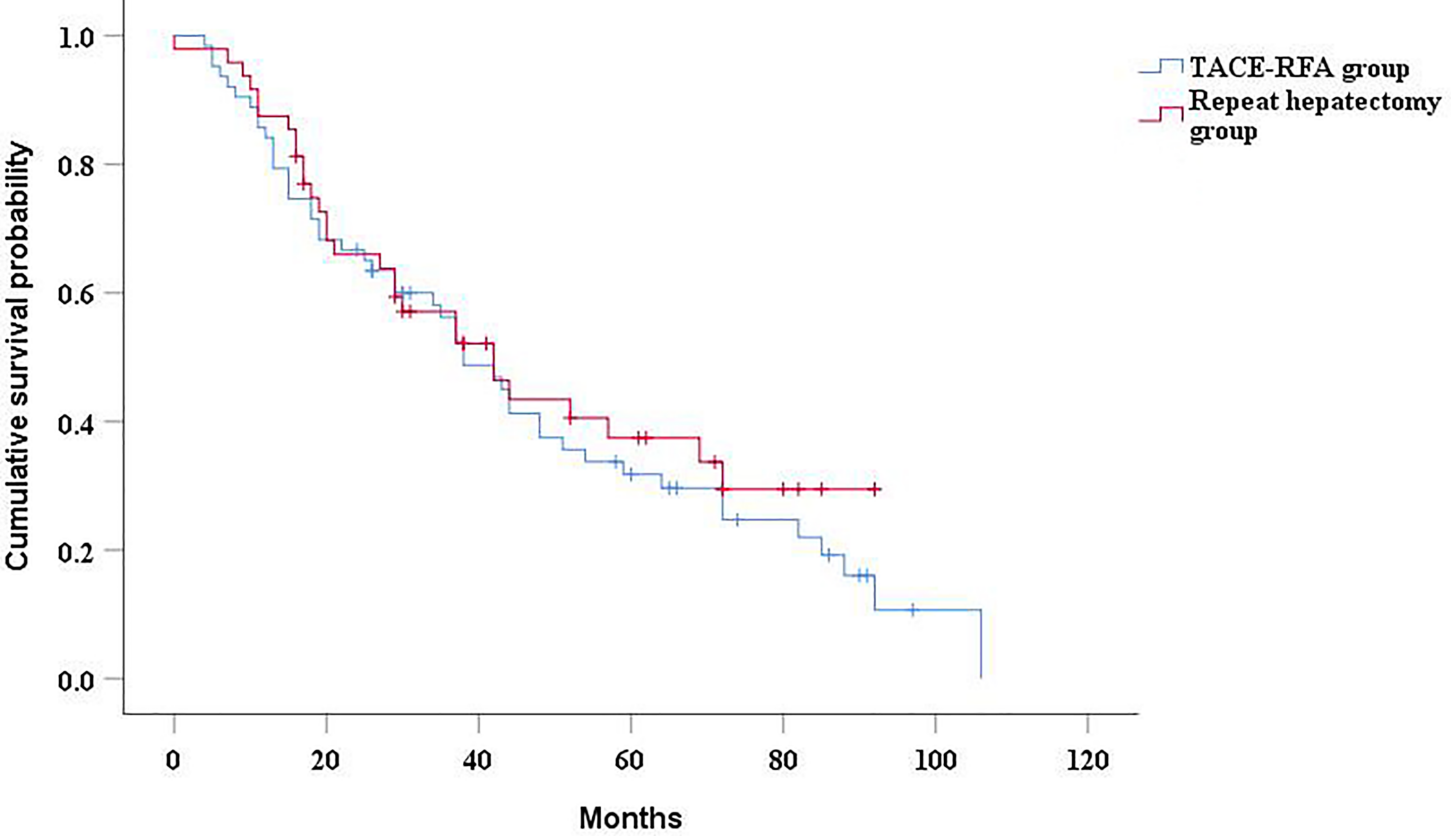
Kaplan-Meier curves of cumulative survival in recurrent HCC patients who received TACE-RFA or repeat hepatectomy.

The median OS was 38 months (95%CI: 28.9 months, 47.1 months) in the TACE-RFA group and 42 months (95%CI: 26.6months, 57.4 months) in the repeat hepatectomy group, with no statistically significant difference between the two groups (*P*= 0.45, **Figure 2**). Although univariate analysis (**Table 2**) revealed that tumor number, α-Fetoprotein level, and Barcelona Clinic Liver Cancer (BCLC) stage were associated with OS, when these three factors were included in multivariate analysis (**Table 3**), none of them was an independent prognostic factor for OS (P>0.05).

**Table 2:** Univariate analysis of prognostic factors for overall survival and progression-free survival.

Variables	OS		PFS	
	HR (95% CI)	*P* value	HR (95% CI)	*P* value
**Gender**				
Male	1		1	
Female	1.058 (0.557, 2.010)	0.863	1.098 (0.580, 2.079)	0.774
**Age (years)**	1.001 (0.983, 1.019)	0.922	1.001 (0.983, 1.019)	0.955
**Bilirubin (µmol/L)**	0.990 (0.966, 1.014)	0.423	0.975 (0.950, 1.002)	0.064
**Albumin (g/L)**	0.992 (0.950, 1.036)	0.715	1.002 (0.962, 1.044)	0.920
**PT (s)**	1.056 (0.943, 1.182)	0.345	1.058 (0.944, 1.187)	0.329
**AST (µmol/L)**	1.007 (0.993, 1.022)	0.306	0.999 (0.984, 1.014)	0.907
**ALT (µmol/L)**	1.003 (0.989, 1.017)	0.686	1.002 (0.988, 1.016)	0.783
**Tumor size**	1.017 (0.943, 1.096)	0.664	1.025 (0.951, 1.104)	0.522
**Tumor number**	1.487 (1.139, 1.942)	0.004	1.625 (1.251, 2.110)	0.000
**Hepatitis**				
Hepatitis B	1		1	
Other	0.839 (0.443, 1.591)	0.591	1.222 (0.672, 2.220)	0.511
**α-Fetoprotein level**				
≥400 ng/mL	1		1	
<400 ng/ml	1.611 (0.999, 2.598)	0.050	1.683 (1.059, 2.674)	0.028
**Child-Pugh score**				
A	1		1	
B	1.223 (0.606, 2.466)	0.574	0.880 (0.453, 1.710)	0.706
**BCLC stage**				
B	1			
A	1.686 (0.940, 3.023)	0.080	1.942 (1.082, 3.421)	0.026
**Interval of recurrence** **from initial treatment** **(months)**	1.000 (0.988, 1.012)	0.970	1.002 (0.990, 1.014)	0.728
**Treatment method**				
Repeat hepatectomy	1		1	
TACE-RFA	1.193 (0.750, 1.897)	0.456	1.113 (0.710, 1.743)	0.640

OS, Overall survival; PFS, Progression-free survival; HR, Hazard ratio; CI, Confidence interval; PT, Prothrombin time; AST, Aspartate aminotransferase; BCLC, Barcelona Clinic Liver Cancer; TACE, Transcatheter arterial chemoembolization; RFA, Radiofrequency ablation.

**Table 3:** Multivariate analysis of prognostic factors for overall survival.

Variables	HR (95% CI)	*P* value
**Tumor number**	1.288 (0.831, 1.996)	0.258
**α-Fetoprotein level**		
>400 ng/mL	1	
≤400 ng/ml	1.673 (0.869, 3.219)	0.123
**BCLC stage**		
B		
A	1.235 (0.570, 2.679)	0.593

HR, Hazard ratio; CI, Confidence interval; BCLC, Barcelona Clinic Liver Cancer.

### Progression-Free Survival

**Figure 3:**
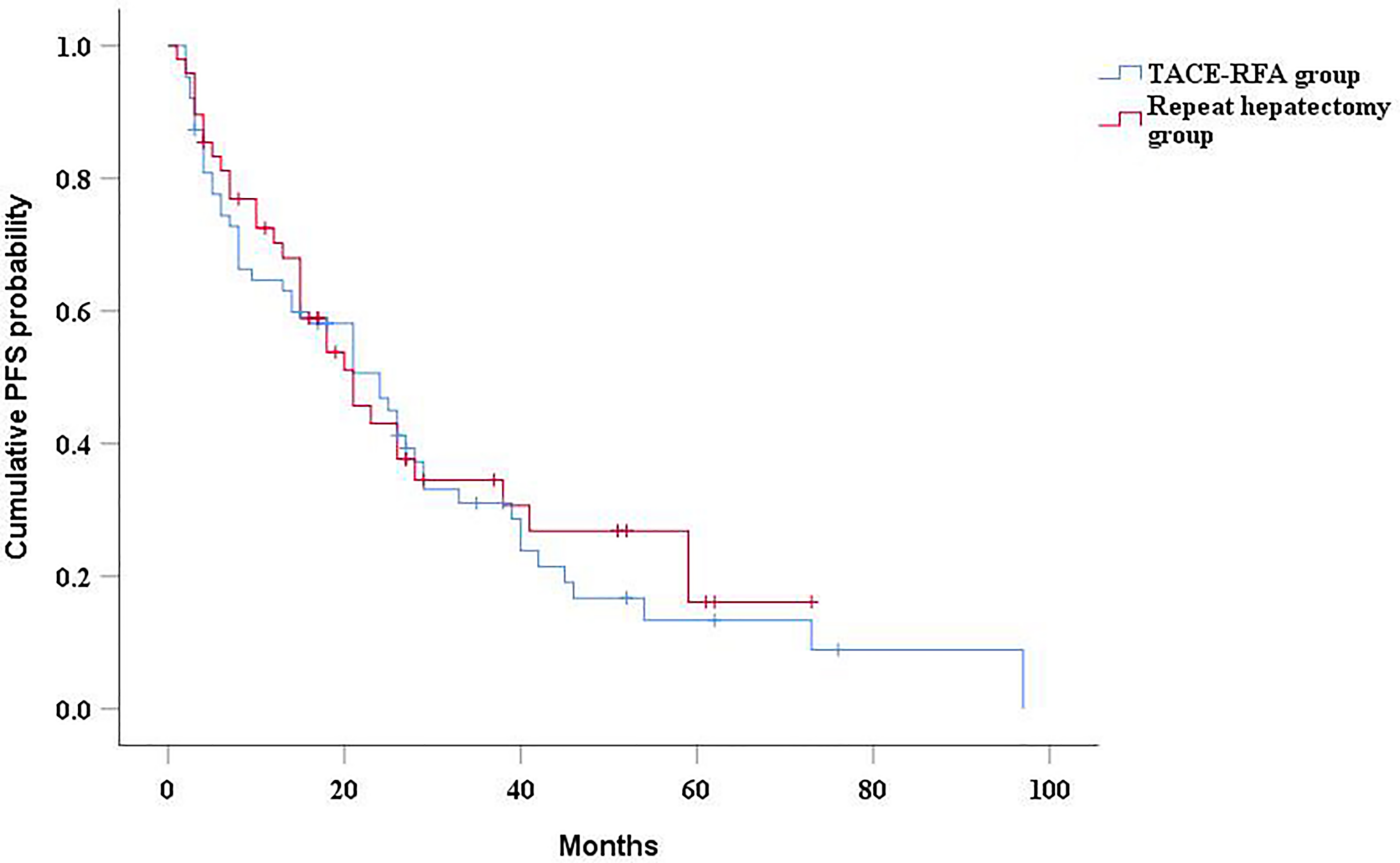
Kaplan-Meier curves of PFS in recurrent HCC patients who received TACE-RFA or repeat hepatectomy.

The median PFS of the TACE-RFA group was 24 months (95%CI: 15.2months, 32.8 months), and the median PFS of the repeat hepatectomy group was 21 months (95%CI: 13.4months, 28.6 months), with no significant difference between the two groups (*P*=0.634) (**Figure 3**). Univariate analysis (**Table 2**) indicated that bilirubin, tumor number, α-Fetoprotein level, and BCLC stage were associated with PFS. These four factors were included in multivariate analysis, and the results demonstrated that tumor number was an independent prognostic factor affecting PFS (**Table 4**).

**Table 4:** Multivariate analysis of prognostic factors for time to progression.

Variables	HR (95% CI)	*P* value
**Bilirubin (µmol/L)**	1.022 (0.987, 1.058)	0.223
**Tumor number**	1.951 (1.246, 3.056)	0.004
**α-Fetoprotein level**		
>400 ng/mL	1	
≤400 ng/ml	1.717 (0.883, 3.338)	0.111
**BCLC stage**		
A		
B	1.014 (0.476, 2.162)	0.971

HR, Hazard ratio; CI, Confidence interval; BCLC, Barcelona Clinic Liver Cancer.

### Subgroup Analysis by Tumor Size

**Figure 4:**
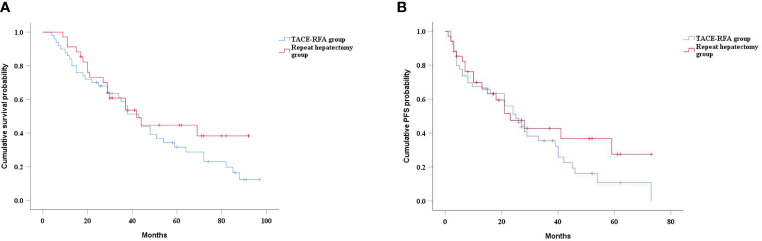
Kaplan-Meier curves of cumulative survival **(A)** and PFS **(B)** in in patients with tumors smaller than 5cm in diameter.

**Figure 5:**
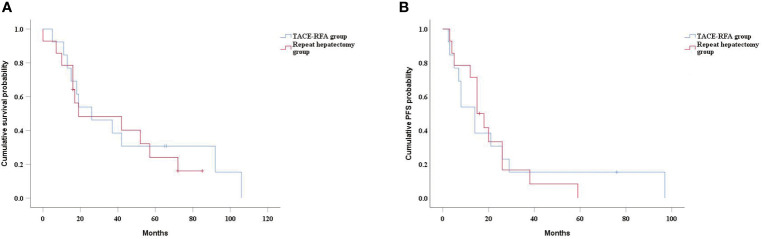
Kaplan-Meier curves of cumulative survival **(A)** and PFS **(B)** in in patients with tumors larger than 5cm in diameter.

In the subgroup analysis, there was no significant difference in median OS between the TACE-RFA group and the repeat hepatectomy group for recurrent HCC patients with tumor diameter less than 5cm (43 months *vs* 42 months, *P*=0.268) (**Figure 4A**). Similarly, there was no statistically significant difference in median PFS between the two groups (25 months *vs* 23 months, *P*=0.27) (**Figure 4B**). There was also no difference in median OS (26 months *vs* 19 months, *P*=0.713) (**Figure 5A**) and PFS (14 months *vs* 15 months, *P*=0.937) (**Figure 5B**) between the two groups for recurrent HCC patients with tumor size larger than 5cm.

### Complications

One patient in the repeat hepatectomy group died of massive hemorrhage after surgery, while no treatment-related death occurred in the TACE-RFA group. In addition, liver failure occurred in 5 patients and gastrointestinal bleeding occurred in 4 patients in the hepatectomy group. The incidence of major complications was higher in the repeat hepatectomy group than in the TACE-RFA group (*P*=0.003) (**Table 5**). Similarly, there was a higher rate of minor complications in the repeat hepatectomy group. Fever and abdominal pain were the most common minor complications, and symptoms improved significantly after symptomatic management.

**Table 5:** Complications after treatment.

Variable	TACE-RFA (N=63) (No, %)	Repeat hepatectomy (N=48) (No, %)	*P* value
**Major complication**	4 (6.3%)	13 (27.1%)	0.003
Mortality	0	1 (2.1%)	
Liver failure	2 (3.2%)	5 (10.4%)	
Gastrointestinal hemorrhage	1 (1.6%)	4 (8.3%)	
Abdominal pain			
Grade 3	1 (1.6%)	2 (4.2%)	
Vomiting			
Grade 3	0	1 (2.1%)	
**Minor complication**			
Fever			
Grade 1	15 (23.8%)	21 (43.8%)	0.026
Grade 2	8 (12.7%)	13 (27.1%)	0.055
Abdominal pain			
Grade 1	20 (31.7%)	27 (56.3%)	0.01
Grade 2	12 (19.0%)	19 (39.6%)	0.017
Vomiting			
Grade 1	11 (17.5%)	16 (33.3%)	0.053
Grade 2	6 (9.5%)	8 (16.7%)	0.261

TACE, Transcatheter arterial chemoembolization; RFA, Radiofrequency ablation.

## Discussion

It has been reported that TACE can reduce hepatic arterial blood flow, thereby reducing heat sink effect and increasing the efficacy of RFA. Meanwhile, TACE can detect satellite lesions, which is beneficial to RFA (19). Hence, the combination of TACE and RFA was supposed to improve survival of recurrent HCC patients. The results of this study indicated that TACE-RFA achieved similar local efficacy and survival outcomes in patients with recurrent HCC compared with repeat hepatectomy, with no significant difference in OS and PFS between the two groups. Therefore, TACE-RFA may be a better choice for recurrent HCC patients who are not suitable for reoperation.

Song et al. retrospectively analyzed the clinical data of patients with recurrent HCC after hepatic resection who received TACE-RFA or TACE alone, and the results showed that TACE-RFA achieved better PFS than patients in the TACE alone group (30). Meanwhile, Peng et al. compared the efficacy of TACE-RFA with repeat hepatectomy in the treatment of recurrent HCC and concluded that TACE-RFA provided comparable OS and PFS compared with repeat hepatectomy (25). Similarly, our results also showed that TACE-RFA can achieve satisfactory results. This suggests that combination therapy, as described by the theoretical advantage, has a synergistic effect and is beneficial for patients with recurrent HCC.

However, in the study of Song (30) and Peng et al. (25), all patients with recurrent HCC had tumor diameters of less than 5cm, while for patients with recurrent HCC with tumor diameters of more than 5cm, no study has reported the efficacy of TACE-RFA and repeat hepatectomy in these patients. In this study, subgroup analysis results indicated that TACE-RFA and repeat hepatectomy had similar OS and PFS for recurrent HCC with tumor diameter greater than 5cm, indicating that TACE-RFA also had a satisfactory effect for recurrent HCC with tumor diameter greater than 5cm.

Although our study also demonstrated that TACE-RFA and repeat hepatectomy had similar therapeutic effects, complications should not be ignored in the choice of treatment modality for patients with recurrent HCC. In this study, the incidence of major complications in the repeat hepatectomy group was significantly higher than that in the TACE-RFA group. It was reported that the incidence of major complications in repeat hepatectomy was 6%-24.4% (31, 32), and the incidence of major complications in this study was 27.1%, slightly higher than the results reported in other studies. This may be because the tumor diameter of some HCC patients in this study was larger than 5cm, and the larger the tumor diameter was, the more likely it was to lead to complications. This also suggested that TACE-RFA may be a safer and less invasive treatment for patients with recurrent HCC.

This study was a retrospective study, so the non-randomized design was a major limitation of the study. Therefore, it is necessary to conduct prospective multicenter randomized controlled trial to verify our results. Meanwhile, no propensity matching analysis was conducted in this study, because the number of patients in this study was limited, and there was no significant difference in baseline data between the two groups.

## Conclusions

In conclusion, compared with repeat hepatectomy, TACE-RFA has the comparable local efficacy and long-term survival results for patients with recurrent HCC after hepatectomy. Meanwhile, TACE-RFA has also achieved satisfactory results for patients with tumor diameter greater than 5cm. In addition, patients in the TACE-RFA group had relatively fewer complications.

## Data Availability Statement

The raw data supporting the conclusions of this article will be made available by the authors, without undue reservation.

## Ethics Statement

The studies involving human participants were reviewed and approved by Union Hospital, Tongji Medical College, Huazhong University of Science and Technology. Written informed consent to participate in this study was provided by the participants’ legal guardian/next of kin.

## Author Contributions

XZ, YR, and HH collected the patients’ data. XZ drafted the manuscript. XZ and KQ revised the manuscript. XZ and YQR analyzed and interpreted the data. XZ and KQ and made substantial contributions to the conception of the work. YR and HH made substantial contributions to the design of the work and have revised the manuscript substantively. All authors contributed to the article and approved the submitted version.

## Funding

This work was supported by the National Natural Sciences Foundation of China (NO. 81701800).

## Conflict of Interest

The authors declare that the research was conducted in the absence of any commercial or financial relationships that could be construed as a potential conflict of interest.

## Publisher’s Note

All claims expressed in this article are solely those of the authors and do not necessarily represent those of their affiliated organizations, or those of the publisher, the editors and the reviewers. Any product that may be evaluated in this article, or claim that may be made by its manufacturer, is not guaranteed or endorsed by the publisher.
